# Real-Life Use of Ceftolozane/Tazobactam for the Treatment of Bloodstream Infection Due to Pseudomonas aeruginosa in Neutropenic Hematologic Patients: a Matched Control Study (ZENITH Study)

**DOI:** 10.1128/spectrum.02292-21

**Published:** 2022-04-27

**Authors:** Alba Bergas, Adaia Albasanz-Puig, Ana Fernández-Cruz, Marina Machado, Andrés Novo, David van Duin, Carolina Garcia-Vidal, Morgan Hakki, Isabel Ruiz-Camps, José Luis del Pozo, Chiara Oltolini, Catherine DeVoe, Lubos Drgona, Oriol Gasch, Malgorzata Mikulska, Pilar Martín-Dávila, Maddalena Peghin, Lourdes Vázquez, Júlia Laporte-Amargós, Xavier Durà-Miralles, Natàlia Pallarès, Eva González-Barca, Ana Álvarez-Uría, Pedro Puerta-Alcalde, Juan Aguilar-Company, Francisco Carmona-Torre, Teresa Daniela Clerici, Sarah B. Doernberg, Lucía Petrikova, Silvia Capilla, Laura Magnasco, Jesús Fortún, Nadia Castaldo, Jordi Carratalà, Carlota Gudiol

**Affiliations:** a Infectious Diseases Department, Bellvitge University Hospital, IDIBELL, University of Barcelona, Barcelona, Spain; b Clinical Microbiology and Infectious Diseases Department, General University Hospital Gregorio Marañón, Instituto de Investigación Sanitaria Gregorio Marañón, Madrid, Spain; c Infectious Disease Unit, Internal Medicine Department, Puerta de Hierro Hospital, Madrid, Spain; d Hematology Department, Son Espases Hospital, Mallorca, Spain; e Division of Infectious Diseases, University of North Carolina, Chapel Hill, North Carolina, USA; f Infectious Diseases Department, Hospital Clínic, Barcelona, Spain; g Division of Infectious Diseases, Oregon Health and Science University, Portland, Oregon, USA; h Infectious Diseases Department, Vall d’Hebron University Hospital, Barcelona, Spain; i Infectious Diseases Division, Clinical Microbiology, Clínica Universidad de Navarra, Immune and Infectious Inflammatory Diseases Research, IdiSNA, Pamplona, Spain; j Unit of Infectious and Tropical Diseases, IRCCS San Raffaele Scientific Institute, Milan, Italy; k Infectious Diseases Division, Department of Medicine, University of California, San Francisco, California, USA; l Oncohematology Department, Comenius University and National Cancer Institute, Bratislava, Slovakia; m Infectious Diseases Department, Parc Taulí University Hospital, Sabadell, Spain; n Division of Infectious Diseases, University of Genova and San Martino Hospital, Genoa, Italy; o Infectious Diseases Department, Ramon y Cajal Hospital, Madrid, Spain; p Infectious Diseases Clinic, Department of Medicine, University of Udine and Azienda Sanitaria Universitaria Integrata, Udine, Italy; q Hematology Service, Hospital Clínico Universitario, Salamanca, Spain; r Statistics Advisory Service, Institute of Biomedical Research of Bellvitge, Barcelona, Spain; s Hematology Department, Institut Català d’Oncologia (ICO), Hospital Duran i Reynals, IDIBELL, University of Barcelona, Barcelona, Spain; t Department of Medical Oncology, Vall d’Hebron University Hospital, Vall d’Hebron Institute of Oncology (VHIO), Barcelona, Spain; u Hematology Department, IRCCS San Raffaele Scientific Institute, Milan, Italy; v Microbiology Department, Parc Taulí University Hospital, Sabadell, Spain; w Centro de Investigación Biomédica en Red de Enfermedades Infecciosas (CIBERINFEC), Instituto de Salud Carlos III, Madrid, Spain; x Institut Català d’Oncologia (ICO), Hospital Duran i Reynals, IDIBELL, Barcelona, Spain; Johns Hopkins Hospital

**Keywords:** multidrug-resistant, *Pseudomonas aeruginosa*, bacteremia, bloodstream infection, neutropenia, hematologic malignancy, ceftolozane/tazobactam

## Abstract

We sought to assess the characteristics and outcomes of neutropenic hematologic patients with Pseudomonas aeruginosa (PA) bloodstream infection (BSI) treated with ceftolozane-tazobactam (C/T). We conducted a multicenter, international, matched-cohort study of PA BSI episodes in neutropenic hematologic patients who received C/T. Controls were patients with PA BSI treated with other antibiotics. Risk factors for overall 7-day and 30-day case fatality rates were analyzed. We compared 44 cases with 88 controls. Overall, 91% of episodes were caused by multidrug-resistant (MDR) strains. An endogenous source was the most frequent BSI origin (35.6%), followed by pneumonia (25.8%). There were no significant differences in patient characteristics between groups. C/T was given empirically in 11 patients and as definitive therapy in 41 patients. Treatment with C/T was associated with less need for mechanical ventilation (13.6% versus 33.3%; *P* = 0.021) and reduced 7-day (6.8% versus 34.1%; *P* = 0.001) and 30-day (22.7% versus 48.9%; *P* = 0.005) mortality. In the multivariate analysis, pneumonia, profound neutropenia, and persistent BSI were independent risk factors for 30-day mortality, whereas lower mortality was found among patients treated with C/T (adjusted OR [aOR] of 0.19; confidence interval [CI] 95% of 0.07 to 0.55; *P* = 0.002). Therapy with C/T was associated with less need for mechanical ventilation and reduced 7-day and 30-day case fatality rates compared to alternative agents in neutropenic hematologic patients with PA BSI.

**IMPORTANCE** Ceftolozane-tazobactam (C/T) has been shown to be a safe and effective alternative for the treatment of difficult to treat infections due to Pseudomonas aeruginosa (PA) in the general nonimmunocompromised population. However, the experience of this agent in immunosuppressed neutropenic patients is very limited. Our study is unique because it is focused on extremely immunosuppressed hematological patients with neutropenia and bloodstream infection (BSI) due to PA (mainly multidrug resistant [MDR]), a scenario which is often associated with very high mortality rates. In our study, we found that the use of C/T for the treatment of MDR PA BSI in hematological neutropenic patients was significantly associated with improved outcomes, and, in addition, it was found to be an independent risk factor associated with increased survival. To date, this is the largest series involving neutropenic hematologic patients with PA BSI treated with C/T.

## INTRODUCTION

Bloodstream infection (BSI) is one of the most frequent infectious complications in hematologic patients with neutropenia and is associated with high morbidity and mortality (1). The epidemiology of BSI in neutropenic patients has changed in the last decades, with Gram-negative bacilli (GNB) as the leading cause of infection in the great majority of institutions (2–6). In this line, Pseudomonas aeruginosa (PA) is one of the three most frequent Gram-negatives, along with Escherichia coli and Klebsiella pneumoniae (7). Importantly, the emergence of antibiotic resistance in PA has become a major clinical problem, since the number of active antibiotics against the multidrug-resistant (MDR) and extensively drug-resistant (XDR) strains is small, and, in addition, the use of some of the active agents, namely, colistin/polymyxin B, aminoglycosides, and fosfomycin, is limited due to relatively poor activity and toxicity issues (8). Moreover, the administration of adequate initial empirical antibiotic therapy in neutropenic cancer patients with PA BSI is crucial, since very poor outcomes have been reported in this high-risk population when adequate empirical treatment is delayed (9–11).

Ceftolozane-tazobactam (C/T) is a combination of an oxyimino-aminothiazolyl cephalosporin and a β-lactamase inhibitor with activity against GNB, including PA, and has a safety profile similar to other cephalosporins (12). C/T is less susceptible to some resistant mechanisms, such as cell efflux and bacterial degradation by several β-lactamases, and displays a 90% activity against PA, including the MDR and XDR strains (13, 14). C/T has been approved for the treatment of complicated urinary tract infections (15), complicated intraabdominal infections (16), and ventilator-associated pneumonia (17). C/T has been used in the general nonimmunocompromised population not only for the approved indications but also for the treatment of other difficult to treat infections (18–22). Nevertheless, data regarding the usefulness of C/T in hematologic patients are particularly scarce (23–26). Published data are limited to a series of 6 hematological patients with infection due to MDR PA (23), a case-control study that includes 19 patients treated with C/T (24), two single case series (25, 26), and a retrospective study involving 69 immunosuppressed patients of whom 18 had hematologic malignancies (27). Although the clinical success rates reported in the aforementioned studies reach >83%, these studies have some limitations. They are retrospective studies with a small number of patients, not all the infections are produced by MDR strains, and the types of infections are diverse. In addition, there are no data focusing on high-risk hematologic patients with neutropenia and PA BSI, which is a clinical scenario that is often associated with very high mortality rates.

The aim of our study was to provide “real-life” data comparing the effectiveness of C/T for the treatment of BSI due to MDR PA in neutropenic hematologic patients with other antibiotics with antipseudomonal activity.

## RESULTS

A total of 44 cases and 88 controls were analyzed. Patient characteristics are shown in Table 1. There were no clinically relevant differences between cases and controls. The rates of infection due to MDR and XDR PA isolates were equal between groups. Adequate initial empirical antibiotic therapy was administered similarly in cases and controls (50% versus 45%, *P* = 0.62). In the cases group, empirical C/T was considered inadequate in 2/11 (18.2%) patients, whereas 20/33 patients (60.6%) were given inadequate empirical therapy with other antibiotics.

**TABLE 1:** Clinical characteristics of patients with Pseudomonas aeruginosa bloodstream infection compared by treatment groups

Characteristics^*a*^	Total *n* = 132 (%)^*b*^	Cases *n* = 44 (% or IQR)^*b*^	Controls *n* = 88 (% or IQR)^*b*^	*P* value
Gender (male)	85 (64.49)	28 (63.6)	57 (64.8)	1.00
Age (yrs, median, IQR)	54 (41–65)	52 (37.2–61.7)	54.5 (41–67.5)	0.68
Comorbidities	47 (35.6)	15 (34.1)	32 (36.4)	0.84
Chronic cardiac disease	18 (13.6)	3 (6.8)	15 (17)	0.17
Diabetes mellitus	11 (8.3)	3 (6.8)	8 (9.1)	0.75
Chronic obstructive pulmonary disease	10 (7.6)	1 (2.3)	9 (10.2)	0.16
Chronic liver disease	7 (5.3)	2 (4.5)	5 (5.7)	1.00
Chronic kidney disease	4 (3)	2 (4.5)	5 (5.7)	0.60
Hematologic malignancy				
Acute myeloid leukemia	67 (50.8)	24 (54.5)	43 (48.9)	0.58
Acute lymphoid leukemia	15 (11.4)	6 (13.6)	9 (10.2)	0.57
Lymphoproliferative disorder	36 (27.3)	10 (22.7)	26 (29.5)	0.53
Chronic lymphocytic leukemia	5 (3.8)	2 (4.5)	3 (3.4)	1.00
Multiple myeloma	5 (3.8)	1 (2.3)	4 (4.5)	0.66
Other	4 (3)	1 (2.3)	3 (3.4)	1.00
Hematopoietic stem cell transplant (HSCT)	49 (37.1)	17 (38.6)	32 (36.4)	0.79
Type of HSCT				
Autologous HSCT	3 (6.2)	0 (0)	3 (9.7)	0.54
Allogeneic HSCT	45 (91.8)	17 (100)	28 (87.5)	0.54
Graft-versus-host disease	14 (32.6)	6 (40)	8 (28.6)	0.50
Uncontrolled disease	55 (45.5)	19 (45.2)	36 (45.6)	0.97
High-risk MASCC score (<21 points)	90 (75.6)	33 (75)	57 (76)	1.00
Profound neutropenia (<0.1 × 10^9^)	84 (64.1)	31 (70.5)	53 (60.9)	0.340
Duration of neutropenia prior to infection (days, median, IQR)	7 (2–15)	8 (2–16)	7 (3–15)	0.910
Duration of neutropenia after infection (days, median, IQR)	5 (2–12)	8 (4–19)	4 (2–9)	0.080
Prior fluoroquinolone prophylaxis (1 mo)	53 (40.2)	25 (56.8)	28 (31.8)	0.006
Prior antibiotic therapy (1 mo)	106 (80.9)	36 (81.8)	79 (80.5)	1.000
Previous corticosteroid therapy (1 mo)	78 (60)	23 (52.3)	55 (64)	0.256
Prior hospital admission (3 mo)	84 (64.1)	31 (70.5)	53 (60.9)	0.337
Prior ICU admission (3 mo)	19 (14.4)	9 (20.5)	10 (11.4)	0.192
Nosocomial acquisition	126 (95.5)	44 (100)	82 (93.2)	0.18
Source of BSI				
Endogenous source	47 (35.6)	13 (29.5)	34 (38.6)	0.40
Pneumonia	34 (25.8)	9 (20.5)	25 (28.4)	0.401
Intravascular catheter infection	14 (10.6)	3 (6.8)	11 (12.5)	0.384
Skin and soft tissue infection	9 (6.8)	5 (11.4)	4 (4.5)	0.15
Urinary tract infection	8 (6.1)	5 (11.4)	3 (3.4)	0.116
Perianal infection	7 (5.3)	3 (6.8)	4 (4.5)	0.686
Mucositis	4 (3)	1 (2.3)	3 (3.4)	1.000
Neutropenic enterocolitis	4 (3)	1 (2.3)	3 (3.4)	1.000
Other	6 (4.5)	4 (9.1)	2 (2.3)	0.09
High-risk BSI	68 (51.5)	25 (56.8)	43 (48.9)	0.461
Polymicrobial BSI	6 (4.6)	2 (4.5)	4 (4.5)	1.000
Septic shock at presentation	42 (32.1)	13 (29.5)	29 (33.3)	0.697
Gangrenous ecthyma	10 (7.6)	8 (18.2)	2 (2.3)	0.002
Multidrug-resistant Pseudomonas aeruginosa	120 (90.9)	40 (90.9)	80 (90.9)	1.000
Extensively resistant P. aeruginosa	44 (33.3)	15 (34.1)	29 (33)	0.89

a BSI, bloodstream infection; MASCC, Multinational Association for Supportive Care in Cancer.

b Qualitative data are expressed as numbers (%), unless otherwise indicated, and quantitative data are expressed as means ± standard deviation (SD) or median and interquartile range (IQR; 25th to 75th percentiles), as appropriate.

### Use of C/T.

The empirical and targeted therapies used in cases and controls are detailed in Table 2. C/T was given empirically in 11 patients (25%). In three patients, it was replaced by another regimen afterward (two because of resistance and one due to susceptibility to other β-lactams), and in eight patients (18.1%), it was continued as targeted therapy. The indication for empirical C/T therapy was mainly previous colonization/infection by MDR PA (8/11; 72.7), followed by septic shock (*n* = 1/11; 9.1%), persistent fever (*n* = 1/11; 9.1%), and unfavorable outcome (*n* = 1/11, 9.1%). Empirical C/T therapy was administered mainly in combination with other antibiotics (*n* = 7/11, 63.6%).

**TABLE 2:** Therapy regimens by treatment group

Treatment type^*a*^	Total *n* = 132 (%)	Cases *n* = 44 (%)	Controls *n* = 88 (%)
Empirical treatment			
Monotherapy	66/132 (50)	18/44 (40.9)	48/88 (54.5)
Ceftolozane-tazobactam	4/66 (6)	4/18 (22.2)	0/48 (0)
Piperacilin/tazobactam	23/66 (34.8)	6/18 (33.3)	17/48 (35.4)
Antipseudomonal carbapenems (meropenem/imipenem)	28/66 (42.4)	6/18 (33.3)	22/48 (45.8)
Antipseudomonal cephalosporins (cefepime/ceftazidime)	8/66 (12.1)	2/18 (11.1)	6/48 (12.5)
Others^*b*^	3/66 (4.5)	0/18 (0)	3/48 (6.2)
Combination therapy	63/132 (47.3)	23/44 (52.3)	40/88 (45.5)
C/T + AG	6/63 (9.5)	6/23 (26.1)	0/40 (0)
C/T + colistin	1/63 (1.6)	1/23 (4.3)	0/40 (0)
Other β-lactam + AG	42/63 (66.7)	13/23 (56.5)	30/40 (75)
Other β-lactam + non-AG	9/63 (14.3)	3/23 (13)	6/40 (15)
Non-β-lactam combination	4/63 (6.3)	0/23 (0)	4/40 (10)
No empirical treatment	3/132 (2.3)	3/44 (6.8)	0/88 (0)
Targeted treatment			
Monotherapy	52/132 (39.4)	17/44 (38.6)	35/88 (39.8)
Ceftolozane-tazobactam	16/52 (30.8)	16/17 (94.1)	0/35 (0)
Piperacilin/tazobactam	8/52 (15.4)	1/17 (5.9)	9/35 (25.7)
Antipseudomonal carbapenems (meropenem, imipenem, doripenem)	7/52 (13.5)	3/17 (0)	10/35 (28.5)
Colistin	8/52 (15.4)	0/17 (0)	8/35 (22.9)
Antipseudomonal cephalosporins (cefepime, ceftazidime)	3/52 (5.8)	0/17 (0)	3/35 (8.5)
Fluoroquinolones	3/52 (5.8)	0/17 (0)	3/35 (8.6)
Amikacin	2/52 (3.8)	0/17 (0)	2/35 (5.7)
Combination therapy (2 antibiotics)	53/132 (40.2)	21/44 (47.7)	32/88 (36.4)
C/T + AG	18/53 (30.1)	14/21 (66.7)	0/32 (0)
C/T + Colistin	5/53 (9.4)	5/21 (23.8)	0/32 (0)
Other β-lactam + AG	19/53 (35.8)	1/21 (4.8)	18/32 (56.2)
β-Lactam + non-AG	8/53 (15.1)	0/21 (0)	8/32 (25)
Non-β-lactam combination	7/53 (13.2)	1/21 (4.8)	6/32 (18.7)
Triple therapy	16/132 (12.1)	6/88 (6.8)^*c*^	10/88 (11.4)
No treatment	11/132 (8.3)	0/44 (0)	11/88 (12.5)

a CT, ceftolozane-tazobactam; AG, aminoglycoside.

b Fluoroquinolone (*n* = 1), amikacin (*n* = 1), nonspecified antibiotic (*n* = 1).

c Two patients received C/T in combination with colistin and fosfomycin.

C/T was used as definitive therapy in 41 patients (93.1%), and in 33 of them (80.5%), it was exclusively used as targeted therapy. The indication for targeted therapy was the identification of an MDR PA isolate in the great majority of patients (*n* = 39/41; 95.1%), whereas it was used in two patients with infection due to a susceptible strain, in one patient due to septic shock, and as a carbapenem-sparing strategy due to suspicion of infection by an extended-spectrum β-lactamase *Enterobacterales* in the remaining patient.

The MIC for C/T was tested in 44 isolates, 41 cases, and 3 controls. Five isolates (11.3%) were resistant to C/T (MIC > 4 mg/L; three cases and two controls). In two of the three cases, C/T was used empirically and afterward was replaced by an active agent. The remaining patient was considered to have intermediate susceptibility (MIC = 8 mg/L), and C/T was used in combination with tobramycin, resulting in a favorable outcome.

The most frequent doses of C/T were 3 g every 8 h (q8h; 2 g ceftolozane and 1 g tazobactam; 25/41, 60.9%), followed by 1.5 g q8h (16/41, 39%). Information regarding the doses was missing in three cases (6.8%). The high doses (3 g q8h) were administered in two patients with pneumonia and in two patients with an endogenous source of BSI. Targeted C/T was administered in extended infusion in six patients using the following doses: 3 g/8 h (*n* = 3) and 0.5 g/8 h (*n* = 1). The dose was not reported for the remaining two cases.

### Antibiotic treatment in the control group.

The combination of a β-lactam plus an aminoglycoside was the most frequently used empirical therapy (*n* = 30/88, 34%), with both antibiotics active in 12 patients (12/29, 41.3%). A β-lactam was the only active drug in 37 patients (37/88, 42%), administered as monotherapy in 18 patients and combined with an aminoglycoside, which had no activity against the PA isolate, in 19 patients.

Targeted therapy with two antibiotics was the most frequent strategy (32/88, 36.3%), and the combination of a β-lactam plus an aminoglycoside was the most commonly used (18/32, 56.2%), whereas monotherapy was used in 35 controls (35/88, 39.8%) mainly with a β-lactam (22/35, 62.8%). Eleven patients did not receive any targeted therapy because of early death.

The resistant rates to the different antibiotic classes are summarized in Table 3.

**TABLE 3:** Resistance rates to the different antibiotic classes

Antibiotic families	Total *n*/available isolates *n* (%)^*a*^	Cases *n*/available isolates *n* (%)^*a*^	Controls *n*/available isolates *n* (%)^*a*^	*P* value
Cephalosporins	95/129 (73.6)	37/44 (84.1)	58/85 (68.2)	0.053
Cefepime	70/103 (68)	34/39 (87.2)	36/64 (56.3)	0.001
Ceftazidime	78/128 (60.9)	28/43 (65.1)	50/85 (58.8)	0.49
Piperacillin-tazobactam	93/127 (73.2)	35/42 (83.3)	58/85 (68.2)	0.071
Carbapenems	82/128 (64.1)	35/43 (81.4)	47/85 (55.3)	0.004
Imipenem	90/122 (73.8)	35/38 (92.1)	55/84 (65.5)	0.002
Meropenem	82/123 (66.7)	35/41 (85.4)	47/82 (57.3)	0.002
Doripenem	15/19 (78.9)	7/8 (87.5)	8/11 (72.7)	0.60
Aztreonam	59/77 (76.6)	26/28 (92.9)	33/49 (67.3)	0.011
Aminoglycosides	74/109 (67.9)	30/41 (73.2)	44/68 (64.7)	0.35
Gentamycin	65/118 (55.1)	28/41 (68.3)	37/77 (48.1)	0.035
Amikacin	31/121 (25.6)	9/37 (24.3)	22/84 (26.2)	0.82
Tobramycin	56/98 (57.1)	25/39 (64.1)	31/59 (52.5)	0.25
Fluoroquinolones	102/129 (79.1)	40/44 (90.9)	62/85 (72.9)	0.017
Ciprofloxacin	95/127 (74.8)	36/42 (85.7)	59/85 (69.4)	0.036
Levofloxacin	58/74 (78.4)	23/25 (92)	35/49 (71.4)	0.042
Fosfomycin	23/48 (47.9)	7/21 (33.3)	16/27 (59.3)	0.074
Colistin	0/113	0/36	0/77	–^*b*^

a *In vitro* susceptibility was determined according to the EUCAST recommendations in all centers from Europe. In the centers from the United States, CLSI breakpoints were used.

b A *P* value was not obtained because there were no cases of colistin resistance in any of the two groups.

### Outcomes.

Table 4 details the outcomes of patients compared by treatment groups. All-cause 7-day and 30-day case fatality rates were significantly lower in cases than in controls. The need for mechanical ventilation was also significantly decreased in cases compared to controls. Although they did not reach statistical significance, the rates of persistent BSI and nephrotoxicity showed a trend toward better outcomes in the group of patients treated with C/T than in patients treated with other antibiotics. The nephrotoxicity reported in the 8 cases was attributed to other antibiotics administered concomitantly or before C/T, mostly amikacin and colistin. Regarding the development of adverse effects, there was only one case of encephalopathy attributed to C/T (2.3%).

**TABLE 4:** Outcomes of 132 patients with Pseudomonas aeruginosa bloodstream infection compared by treatment groups

Endpoints^*a*^	Total *n* = 132 (%)	Cases *n* = 44 (%)	Controls *n* = 88 (%)	*P* value
Primary endpoint				
Seven-day case fatality rate	32 (24.2)	3 (6.8)	29 (34.1)	0.001
Thirty-day case fatality rate	53 (40.2)	10 (22.7)	43 (48.9)	0.005
Secondary endpoints				
Persistent BSI	22 (17.1)	4 (9.1)	18 (21.2)	0.084
ICU admission^*b*^	46 (34.8)	12 (27.3)	34 (38.6)	0.246
Need for invasive mechanical ventilation^*b*^	35 (26.7)	6 (13.6)	29 (33.3)	0.021
Other				
Nephrotoxicity	33 (27.9)	8 (18.2)	25 (32.9)	0.082

a BSI, bloodstream infection; ICU, intensive care unit.

b None of the patients were in the ICU at BSI onset.

Tables 5 and 6 summarize the risk factors associated with 7-day and 30-day case fatality rates. In the multivariate analysis, inadequate empirical antibiotic therapy was identified as an independent risk factor for 7-day case fatality rate, whereas therapy with C/T was associated with increased survival. Pneumonia as the source of BSI, profound neutropenia, and persistent BSI were associated with higher 30-day case fatality rate, whereas treatment with C/T was identified as a mortality protective factor.

**TABLE 5:** Univariate and multivariate analysis of factors associated with 7-day case fatality rate

Characteristics	Dead *n* = 32 (%)	Alive *n* = 100 (%)	*P* value	Adjusted OR (95% CI)^*a*^	*P* value^*b*^
Male gender	22 (68.8)	63 (63)	0.55	0.67 (0.24–1.90)	0.462
Age (yrs) (median, IQR)	55 (18–79)	54 (18–90)	0.73	0.60 (0.2–1.60)	0.309
Inadequate empirical antibiotic therapy	21 (63.6)	41 (41.4)	0.027	2.73 (1.11–6.68)	**0.028**
Therapy with ceftolozane-tazobactam	3 (9.4)	41 (41)	0.001	0.16 (0.04–0.58)	**0.006**
Persistent bloodstream infection	9 (30)	13 (13.1)	0.031	2.13 (0.73–6.21)	0.16

a OR, odds ratio; 95% CI, 95% confidence interval.

b Bold formatting indicates statistical significance.

**TABLE 6:** Univariate and multivariate analysis of factors associated with 30-day case fatality rate

Characteristics^*a*^	Dead *n* = 53 (%)	Alive *n* = 98 (%)	*P* value	Adjusted OR (95% CI)^*a*^	*P* value^*b*^
Female gender	19 (40.4)	28 (59.6)	0.96	0.97 (0.38–2.45)	0.958
Age (yrs) (median, IQR)	53 (18–90)	54.5 (18–79)	0.79	0.98 (0.95–1.00)	0.133
Pneumonia	20 (58.8)	14 (41.2)	0.014	5.45 (1.84–16.13)	**0.002**
Therapy with ceftolozane-tazobactam	10 (22.7)	34 (77.3)	0.004	0.19 (0.07–0.55)	**0.002**
Persistent bloodstream infection	14 (63.6)	8 (36.4)	0.009	5.44 (1.61–18.31)	**0.006**
Infection due to XDR PA	23 (52.3)	21 (47.7)	0.045	1.76 (0.68–4.54)	0.240
Profound neutropenia (<100 cells/mm^3^)	41 (48.8)	43 (51.2)	0.009	5.49 (1.96–0.15.36)	**0.001**

a XDR PA, extensively drug-resistant Pseudomonas aeruginosa; OR, odds ratio; 95% CI, 95% confidence interval.

b Bold formatting indicates statistical significance.

**FIG 1:**
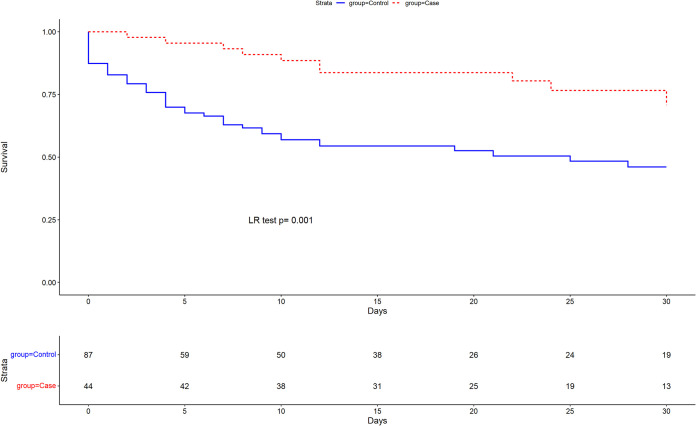
Kaplan-Meier survival curves at 30-day follow-up compared by treatment groups; LR, log rank.

Figure 1 shows the Kaplan-Meier survival curves of patients treated with C/T compared to other antibiotics.

The results of the sensitivity analysis excluding the 11 patients in the control group that did not receive targeted therapy and presented early death were similar (Tables S1 and S2 and Fig. S1 and S2 in the supplemental material).

## DISCUSSION

In our “real-life” experience, we observed that the use of C/T for the treatment of BSI due to MDR PA in high-risk hematological patients with neutropenia was associated with improved outcomes in comparison with other therapeutic alternatives, and it was also found to be an independent risk factor associated with increased survival. To date, this is the largest series involving hematologic patients treated with C/T, and it is particularly relevant because it focuses on neutropenic patients with BSI, a clinical scenario that is often associated with poor outcomes. In addition, more than 25% of the patients in our cohort had pneumonia as the primary source of PA BSI, which is the most life-threatening bacterial infectious complication in neutropenic patients (28).

While real-life experiences of C/T in the general population have been published, data on its use in hematologic patients remain scarce (23–26). Hakki et al. reported a clinical success rate of 83.3% in 6 hematologic patients with MDR PA infections treated with C/T (23). Fernández-Cruz et al. published a case-control study in which 19 patients treated with C/T were compared with 38 patients who received other active regimens (24). Even though there were no differences in the rates of clinical cure at day 14 or in recurrence between the two groups, the case fatality rate at 30-day follow-up was significantly lower among cases (5.3% versus 28.9%, *P* = 0.045) (24). Although pneumonia was the most frequent infection, only 52% of the patients had BSI, and 60% were neutropenic. In addition, the controls were presumably not matched, and, consequently, cases had more frequent infection due to MDR PA strains and had fewer BSIs. Two more single-case series of pediatric neutropenic patients with BSI due to MDR PA reported favorable outcomes (25, 26), although in one of them, the PA strain had become resistant to C/T (26). Interestingly, in this later report, the authors speculated that the resistance mechanism was due to AmpC overexpression, and a synergy effect was observed between C/T and tobramycin, a combination that finally managed to clear the BSI.

We observed significantly improved outcomes in patients treated with C/T compared with controls, including case fatality rates assessed at 7 and 30 days and the need for invasive mechanical ventilation. Of note, the case fatality rates observed in the group of patients who received C/T was unexpectedly low considering the high risk of severe sepsis and death associated with severe PA infections in neutropenic patients, particularly when presenting with pneumonia (28). In line with our results, the previously mentioned case-control study reported even lower 30-day mortality rates (5.3%) in patients treated with C/T (24). As previously noted, in that study, not all patients were neutropenic nor had BSI, only 15.8% fulfilled the criteria for sepsis (while in our cohort, 32.1% of patients presented with septic shock), and a substantial number of infections would be considered low-risk infections.

Persistent BSI was remarkably frequent in our cohort (17.1%), and it was not significantly more common in controls than in cases. Of note, three patients in the control group had catheter-related BSI, but the catheter was removed in two of them. In addition, none of the cases with persistent BSI had developed resistance to C/T. The presence of neutropenia in the setting of severe infections probably played an important role by hindering the clearance of PA from the bloodstream.

Importantly, nephrotoxicity was also more frequent in the control group, although it did not reach statistical significance. The higher rates of kidney injury in the control group were probably associated with an increased use of potentially nephrotoxic antibiotics, such as aminoglycosides and colistin. In addition, the nephrotoxicity reported in patients treated with C/T was attributed to the concomitant or prior use of other nephrotoxic agents, namely, aminoglycosides and colistin.

Unlike other studies that have reported the development of resistance to C/T during therapy (19, 23, 29, 30), in our study, we did not observe this event. In Fernández-Cruz’s study, they also did not find any cases of resistance (24).

In our cohort, treatment with C/T was identified as a protective factor against mortality. Due to the small number of patients who received this drug empirically, we could not conclude that empirical use is also significantly protective, but it is reasonable to speculate that when used promptly in patients at risk for MDR PA infections, it should have a relevant impact on outcomes. The optimal doses of C/T for the treatment of severe PA infections in neutropenic patients and the need for combination therapy have yet to be defined.

Overall, C/T was well tolerated, and only one case of toxicity was reported. This consisted of a patient with normal renal function who developed mild encephalopathy while receiving 2 g q8h of C/T. The patient had a favorable outcome, with a rapid normalization of mental status once the drug was discontinued. Encephalopathy due to cephalosporins, and particularly cefepime, is a well-described adverse event that mainly occurs in patients with impaired renal function (31). To a much lesser extent, this complication in the setting of treatment with C/T has already been reported (32). In individuals with risk factors for neurotoxicity, such as renal insufficiency, therapeutic drug monitoring should be considered.

The results were obtained from a mixed data set (prospectively collected cases matched with retrospectively collected controls). This design allowed us to include and match a large number of patients and should not affect data assessment and analysis because the primary and secondary outcomes were objective endpoints and were fully collected for all patients. Even though the study is not a randomized clinical trial, it provides valuable information about the real use of C/T in daily clinical practice (“real-life”) in a unique setting. Cases and controls were matched according to the multidrug resistance profile of the PA isolate but not by the specific mechanism of resistance; thus, our results have to be interpreted cautiously.

The main strength of the present study is the large number of participating centers from four countries around the world, allowing for the collection of a substantial number of high-risk hematological neutropenic patients with MDR PA BSI treated with C/T and the acquisition of more generalizable results. This study has some limitations that should be acknowledged. First, some of the cases and all the controls were retrospectively collected, which leads to a risk of unmeasured variables and residual confounding. Nevertheless, the primary and secondary endpoints assessed in this study were objective in nature and were collected completely for all cases and controls. Second, the determination of susceptibility/resistance to C/T was assessed by different definitions (EUCAST or CLSI) depending on the country. Third, data regarding the specific mechanisms of resistance to PA for the MDR isolates were not provided. Fourth, follow-up blood cultures were obtained according to each clinician’s criteria and not systematically at 48 h of BSI onset, which could lead to bias. Fifth, a comorbidity index score was not included as an adjusting factor in the multivariate model; however, we found no differences in the comorbidity rates between groups, and, in addition, the Multinational Association for Supportive Care in Cancer (MASCC) risk index score was used as an adjusting factor in the multivariate model, which is a validated tool to measure risk of complications in patients with fever and neutropenia. Sixth, this was not a randomized clinical trial; thus, the choice of therapy could be influenced by several patient-related variables and clinical presentation. Nevertheless, in order to balance patients’ characteristics, we performed a matched control study taking into account the most relevant clinical features. Seventh, 11 patients in the control arm had an early death and did not have the chance to receive targeted C/T therapy. However, the results did not change after performing a sensitivity analysis excluding these 11 controls.

In conclusion, in these real-life data from an observational cohort study of high-risk neutropenic hematologic patients with PA BSI, mostly due to MDR PA strains, therapy with C/T was associated with better outcomes with less need for mechanical ventilation and reduced overall 30-day and 7-day case fatality rates. The empirical use of this agent in febrile neutropenia is highly recommended in patients with risk factors for infection by MDR PA strains, and it should be promptly used in patients with documented infections. The optimal doses of C/T and the need for combination therapy have yet to be established. Further controlled studies involving larger populations are needed.

## MATERIALS AND METHODS

### Study design and setting.

This was a prospective/retrospective, international, multicenter matched control study of neutropenic hematologic patients with BSI due to PA from 1 January 2016 to 30 June 2020 across 17 centers from 4 countries: Spain, United States, Italy, and Croatia. Detailed information regarding the participating centers is provided in the supplemental material. The cases that occurred before the study was designed and approved were collected retrospectively (*n* = 132), whereas those identified after the study was approved were collected prospectively (*n* = 12). All the controls were retrospectively collected and were matched from the IRONIC database (33).

Cases were defined as adult neutropenic hematological patients, including hematopoietic stem cell transplant (HSCT) recipients, with BSI due to PA who received at least 48 h of C/T as empirical or definitive therapy. Controls included adult neutropenic hematological patients and/or HSCT recipients with PA BSI treated with other antibiotics with activity against PA for at least 48 h. Two controls for each case (2:1) were selected from the IRONIC database (33).

### Endpoints.

The primary endpoint was the case fatality rate assessed at 7 and 30 days from BSI onset. The secondary endpoints were the rates of persistent BSI and the need for intensive care unit (ICU) admission and mechanical ventilation only in patients who were not in the ICU and who did not require mechanical ventilation at BSI onset, respectively. BSI was considered to be persistent if the blood cultures were positive after 48 h of adequate antibiotic therapy.

### Variables.

Data regarding baseline characteristics, clinical and microbiological features, and outcomes were carefully collected. Antimicrobial therapy administered before susceptibility results was considered empirical therapy. Empirical antibiotic therapy was considered adequate when it included at least one antibiotic active *in vitro* against the PA strain causing the infection. The concurrent use of ≥2 antibiotics was considered combination treatment. Adequate empirical/targeted combination treatment implied the association of ≥2 *in vitro* active antibiotics. If an empirical combination treatment was administered, including ≥2 antibiotics but only one showed activity against the causative PA strain, it was considered appropriate empirical monotherapy. Inadequate treatment was defined as empirical treatment that did not include any antibiotic with *in vitro* activity. Targeted therapies included those that were administered after the availability of antimicrobial susceptibility testing results within 7 days from BSI onset.

### Definitions.

Definitions are provided in the supplemental material.

### Microbiological studies.

Clinical samples were processed at the microbiology laboratories of each participating center in accordance with standard operating procedures. PA was identified using standard microbiological techniques at each center. *In vitro* susceptibility was determined according to the EUCAST recommendations in all centers from Europe (34). In the centers from the United States, CLSI breakpoints were used (35). We divided the susceptibility profile results into two groups: (i) susceptible strains by both EUCAST and CLSI criteria and (ii) not susceptible strains, which included the “resistant” strains by EUCAST and the “intermediate” and “resistant” strains by CLSI criteria. PA isolate phenotypes were classified in accordance with recent standard definitions (36).

### Statistical analysis.

To define the cohort’s characteristics, categorical variables were presented as the number of cases and percentages, while continuous variables were presented as the mean and standard deviation (SD) or median and interquartile range (IQR). Controls were matched (2:1) according to the closest date of BSI, underlying disease, polymicrobial infection, and susceptibility profile of the PA isolate (susceptible, MDR, or XDR). The controls were randomly selected using the R package optmatch to reduce selection bias.

A logistic regression model was used to estimate the adjusted effect of the intervention on 7-day and 30-day case fatality rates and to identify the main associated clinical factors. Multivariate analysis was performed with variables considered clinically relevant for this study. Statistical analyses were performed using SPSS and R software 4.1.0 (https://cran.r-project.org). A sensitivity analysis was performed excluding the patients in the control group that did not receive targeted therapy.

Mortality survival function of patients treated with C/T or other antibiotics at 30 days from BSI onset was estimated using the Kaplan-Meier curve and compared using a log rank test.

### Ethics.

The study was approved by the institutional review board at Bellvitge University Hospital (reference number EPA031/18) and by the local Research Ethics Committees of participating centers, and it was conducted according to the Declaration of Helsinki guidelines. The need for informed consent was waived by the Clinical Research Ethics Committee for the retrospective cases. The study results are reported following the strengthening the reporting of observational studies in epidemiology (STROBE) recommendations (37).
