# Supplementary material for: Real-Life Use of Ceftolozane/Tazobactam for the Treatment of Bloodstream Infection Due to Pseudomonas aeruginosa in Neutropenic Hematologic Patients: a Matched Control Study (ZENITH Study)
Source: Microbiol Spectr. 2022 Apr 27;10(3):e02292-21. doi: 10.1128/spectrum.02292-21 (PMC9241913; doi:10.1128/spectrum.02292-21)

SUPPLEMENTARY MATERIAL

Participating centers in the ZENITH study and number of patients provided by center

Spain (n=32)

Bellvitge University Hospital, Barcelona (n=2)

Vall d'Hebron University Hospital, Barcelona (n=2)

Hospital Clinic Hospital, Barcelona (n=3)

Parc Taulí Hospital, Barcelona (n=1)

Gregorio Marañon University Hospital, Madrid (n=13)

Puerta de Hierro University Hospital, Madrid (n=2)

Salamanca University Hospital, Castilla la Mancha (n=1)

Ramón y Cajal University Hospital (n=1)

Hospital Son Espases, Mallorca (n=5)

Clínica Universidad de Navarra (n=2)

USA (n=7)

University of North Carolina, Chapel Hill, North Carolina, USA (n=4)

UCSF Medical Center, San Francisco, California, USA (n=1)

Oregon Health and Science University, Portland, Oregon, USA (n=2)

ITALY (n=4)

San Raffaele Scientific Institute, Milan (n=2)

San Martino Hospital, Genova (n=1)

Santa Maria Misericordia University Hospital, Udine (n=1)

SLOVAKIA (n=1)

Comenius University and National Cancer Institute, Bratislava, (n=1)

DEFINITIONS

Neutropenia and profound neutropenia were defined as an absolute neutrophil count <0.5x10^9/L and <0.1x10^9/L, respectively. BSI was considered to be persistent if the blood cultures were positive after 48 hours of adequate antibiotic therapy. Comorbidities were defined as the presence of one or more of the following diseases: chronic obstructive pulmonary disease, heart disease, hepatic disease, diabetes mellitus, chronic kidney disease, and cerebrovascular disease, conjunctive tissue disease, AIDS, immunodeficiency, solid organ transplant, thyroid disease, and inflammatory bowel disease. Uncontrolled disease was considered when patient was refractory to treatment, in situation of relapsed disease, or in course of palliative chemotherapy. The Multinational Association for Supportive Care in Cancer (MASCC) score was calculated as described elsewhere [1].

BSI sources were established using standard US Centres for Disease Control and Prevention criteria for secondary BSI [2]. We defined an endogenous source of BSI in neutropenic patients with absent or mild gastrointestinal symptoms, in whom gut translocation was suspected. Neutropenic enterocolitis was defined in patients with severe (grade III-IV) extensive mucositis, involving the upper and lower gastrointestinal tract. Mucositis was considered in patients with ulcerative lesions involving only the oral cavity. Low-risk BSI was considered when originating in the urinary tract, vascular catheter infection or endogenous source, and high-risk when originating from other sources. Previous corticosteroid treatment was defined as the administration of ≥ 20mg of prednisone, or equivalent dosing, for at least 4 weeks within the last 30 days from BSI onset. Acute kidney injury and nephrotoxicity (AKI) was defined and classified following the AKIN criteria [3].

Early case-fatality rates were defined as death from any cause within 7 days of BSI onset. Overall 30-day case-fatality rate was defined as death from any cause within 30 days of BSI onset.

References:

[1] Klastersky J, Paesmans M, Rubenstein EB et al. The Multinational Association for Supportive Care in Cancer risk index: A multinational scoring system for identifying low-risk febrile neutropenic cancer patients. J Clin Oncol **2000**; 18: 3038-3051]

[2] Garner JS, Jarvis WR, Emori TG, Horan TC, Hughes JM. CDC definitions for nosocomial infections. Am J Infect Control. **1988**; 16:128–140

[3] Khwaja A. KDIGO Clinical Practice Guideline for Acute Kidney Injury. Nephron Clin Pract. **2012**; 120: c179-184. doi: 10.1159/000339789

Table S1. Multivariate Cox analysis of factors associated with 7-day case-fatality *(sensitivity analysis excluding 11 controls that presented an early death)*

	Adjusted OR (95% CI)	p-value
Male gender	1.18 (0.48-2.90)	0.723
Age (years, median, IQR)	0.99 (0.96-1.01)	0.289
Pneumonia	2.30 (0.95-5.56)	0.064
Therapy with ceftolozane/tazobactam	0.23 (0.07-0.79)	0.019
Inadequate empirical antibiotic treatment	2.13 (0.87-5.25)	0.100
Persistent bloodstream infection	1.05 (0.37-3.01)	0.921
Profound neutropenia (<100 cells/mm^3)	5.01 (1.48-16.91)	0.010

Table S2. Multivariate Cox analysis of factors associated with 30-day case-fatality *(sensitivity analysis excluding 11 controls that presented an early death)*

	Adjusted OR (95% CI)	p-value
Male gender	0.90 (0.45-1.78)	0.761
Age (years, median, IQR)	0.98 (0.96-1.01)	0.139
Pneumonia	2.97 (1.47-6.00)	0.003
Therapy with ceftolozane/tazobactam	0.35 (0.15-0.81)	0.015
Inadequate empirical antibiotic treatment	1-10 (0.55-2.22)	0.786
Persistent bloodstream infection	1.50 (0.67-3.35)	0.327
Profound neutropenia (<100 cells/mm^3)	4.42 (1.81-10.76)	0.001

Fig. S1.- Kaplan Meyer survival analysis for the 7-day case-fatality rate *(sensitivity analysis excluding 11 controls that presented an early death)*

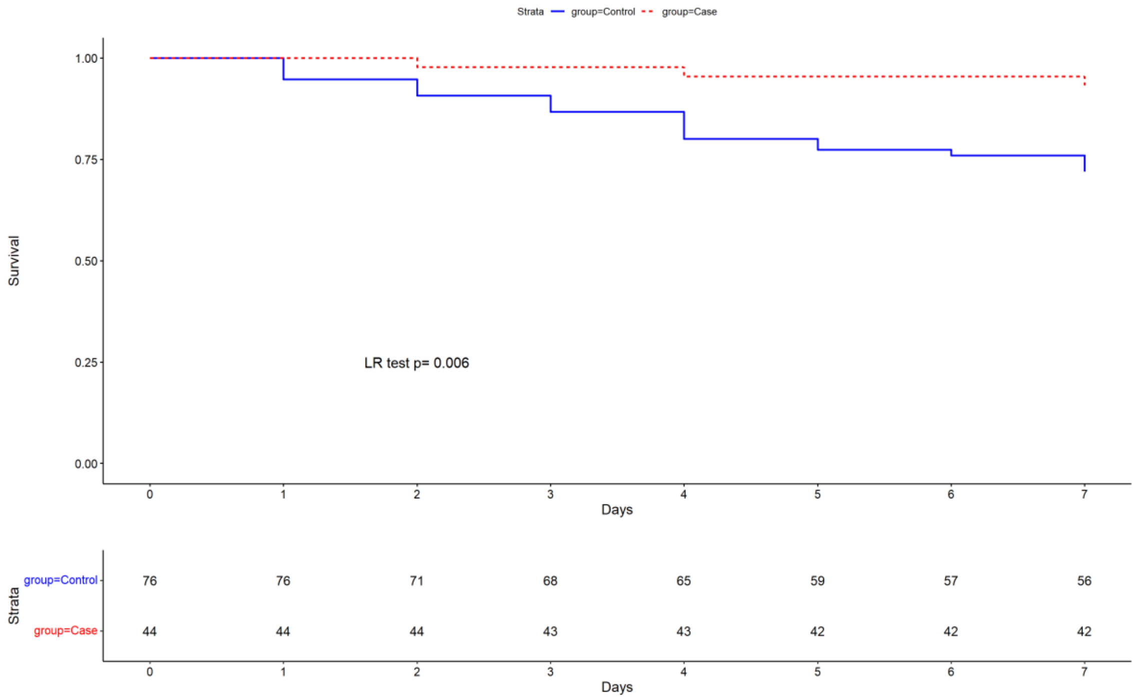

Fig. S2.- Kaplan Meyer survival analysis for the 30-day case-fatality rate *(sensitivity analysis excluding 11 controls that presented an early death)*

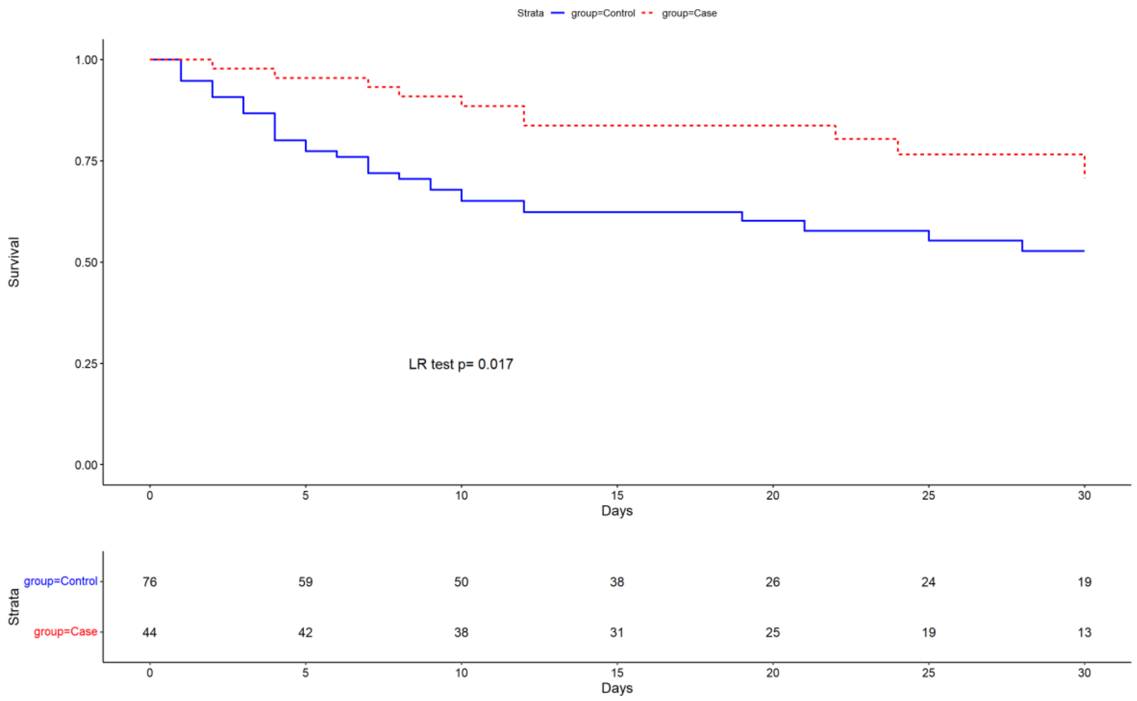

Supplement: SUPPLEMENTAL FILE 1 — Supplemental material. Download spectrum.02292-21-s001.pdf, PDF file, 0.4 MB [file spectrum.02292-21-s001.pdf]
